# Effect of forest fire prevention treatments on bacterial communities associated with productive *Boletus edulis* sites

**DOI:** 10.1111/1751-7915.13395

**Published:** 2019-04-15

**Authors:** Olaya Mediavilla, József Geml, Jaime Olaizola, Juan Andrés Oria‐de‐Rueda, Petr Baldrian, Pablo Martín‐Pinto

**Affiliations:** ^1^ Fire and Applied Mycology Laboratory Departments of Agroforestry Sciences and Vegetal Production and Natural Resources Sustainable Forest Management Research Institute University of Valladolid (Palencia) Avda. Madrid 44 34071 Palencia Spain; ^2^ Biodiversity Dynamics Research Group Naturalis Biodiversity Center Vondellaan 55, PO Box 9517 2300 RA Leiden The Netherlands; ^3^ IDForest‐Biotecnología Forestal Aplicada Calle Curtidores, 17 34004 Palencia Spain; ^4^ Laboratory of Environmental Microbiology Institute of Microbiology of the CAS Vídeňská 1083 14220 Praha 4 Czech Republic

## Abstract

*Cistus ladanifer* scrublands, traditionally considered as unproductive, have nonetheless been observed to produce large quantities of king bolete (*Boletus edulis*) fruitbodies. These pyrophytic scrublands are prone to wildfires, which severely affect fungi, hence the need for fire prevention in producing *C. ladanifer* scrublands. In addition, *B. edulis* productions have severely decreased in the last years. A deeper understanding of the *B. edulis* life cycle and of biotic and abiotic factors influencing sporocarp formation is needed to implement management practices that facilitate *B. edulis* production. For example, some bacteria likely are involved in sporocarp production, representing a key part in the triple symbiosis (plant–fungus–bacteria). In this study, we used soil DNA metabarcoding in *C. ladanifer* scrublands to (i) assess the effect of site history and fire prevention treatment on bacterial richness and community composition; (ii) test if there was any correlation between various taxonomic groups of bacteria and mycelial biomass and sporocarp production of *B. edulis*; and to (iii) identify indicator bacteria associated with the most productive *B. edulis* sites. Our results show that site history drives bacterial richness and community composition, while fire prevention treatments have a weaker, but still detectable effect, particularly in the senescent plots. Sporocarp production correlated positively with genera in Verrucomicrobia. Several genera, e.g. *Azospirillum* and *Gemmatimonas*, were identified as indicators of the most productive sites, suggesting a potential biological role in *B. edulis* fructification. This study provides a better understanding of the triple symbiosis (plant–fungus–bacteria) involved in *C*. *ladanifer*–*B. edulis* systems.

## Introduction

*Cistus ladanifer* L. scrublands, traditionally considered as unproductive ecosystems (Oria‐de‐Rueda *et al*., 2008), are, nonetheless, able to sustain a high diversity of valuable edible mushrooms species (Comandini *et al*., 2006). The most appreciated among these is *Boletus edulis* Bull. that can produce extraordinary amount of fruitbodies in *C. ladanifer* scrublands (Oria‐de‐Rueda *et al*., 2008; Hernández‐Rodríguez *et al*., 2015b). This species is highly sought after as a gourmet food, which generates an important worldwide market (Dentinger *et al*., 2010; Catcheside and Catcheside, 2012).

However, these ecosystems, and the fungal communities associated with them, are frequently affected by wildfires (Martín‐Pinto *et al*., 2006), which is detrimental to the majority of these fungi (Jiménez‐Esquilín *et al*., 2007). This fact has lead to the need for fire prevention treatments, which could improve mushroom production levels (Savoie and Largeteau, 2011).

In addition, due to the severe drought suffered in the Mediterranean basin, *B. edulis* production has plummeted since autumn 2014, with near zero production in some areas in Spain in the autumn of 2017 (O. Mediavilla, personal observation). This dramatic situation has highlighted the need for a deeper understanding of the *B. edulis* life cycle and the environmental factors and biological interactions that influence sporocarp formation.

Besides abiotic environmental factors, biotic factors, such as plant physiology and interactions with other soil fungi and bacteria, are also likely to be involved in sporocarp production (Antony‐Babu *et al*., 2013). A greater understanding of bacterial communities in forest soils and the effect of different forest management options is essential because these microorganisms represent a key part in the triple symbiosis (plant–fungus–bacteria) (Barbieri *et al*., 2005; Bonfante and Anca, 2009), and they represent the most abundant microorganisms in forest soils (Hardoim *et al*., 2015; Baldrian, 2017). Despite this fact, to date, bacteria in forest soils have received less attention than fungi. Bacteria are ubiquitous microbes in many environments, where they may have several symbiotic functions in mushrooms. Some bacterial species play essential roles in nutrient cycling (Pent *et al*., 2017), mediating multiple critical steps in the nitrogen cycle, including N fixation (Lladó *et al*., 2017), which has a strong influence on the abundance of ectomycorrhizal fungi (Frey *et al*., 2004; Allison *et al*., 2007). Other bacterial species take part in C cycling processes (Fierer *et al*., 2013), increasing the availability of carbon for the plant and for the associated fungi that obtain their carbon source from other organisms (Honrubia, 2009). Some groups of bacteria can act also as mycorrhiza helper bacteria (MHB), which are able to stimulate the development of mycorrhiza, or to the contrary, act as pathogen inhibitors and antagonists (Frey‐Klett *et al*., 2007). In this context, previous studies have focused on the role of bacteria in the production of fruiting bodies of other edible fungal species, such as truffles (Splivallo *et al*., 2015).

The symbiotic development of mycorrhizal fungi on plant roots has been reported to be influenced by bacteria present in the mycorrhizosphere (Barbieri *et al*., 2005). Although the role of bacteria in the formation of *B. edulis* mycorrhiza has already been demonstrated (Mediavilla *et al*., 2016), a deeper understanding of the effect of natural bacterial communities on the mycelium in the soil and on sporocarp production is needed.

To date, there is no information about bacterial communities present in the mycorrhizosphere of the *C. ladanifer*–*B. edulis* symbiosis and the possible influences of management treatments. We hypothesize that bacterial communities are affected by site history and fire prevention treatments in these ecosystems. The bacteria observed could play an active role in the presence and fructification of *B. edulis*, and some of them may even act as indicator species at the most productive *B. edulis* sites. Therefore, the aim of this study was to analyse the diversity of bacterial communities and their interaction with *B. edulis* and their potential role in *B. edulis* sporocarp production under different forest fire prevention treatments. Our specific aims were (i) to investigate the effects of different fire prevention treatments and site history on the bacterial richness, abundance and composition; (ii) to determine whether there was a correlation between bacterial communities and *B. edulis* sporocarp production and mycelium present in the soil; and (iii) to identify bacterial indicator species associated with the most productive *B. edulis* sites.

## Results

### Taxonomic composition of the microbial community

**Figure 1:**
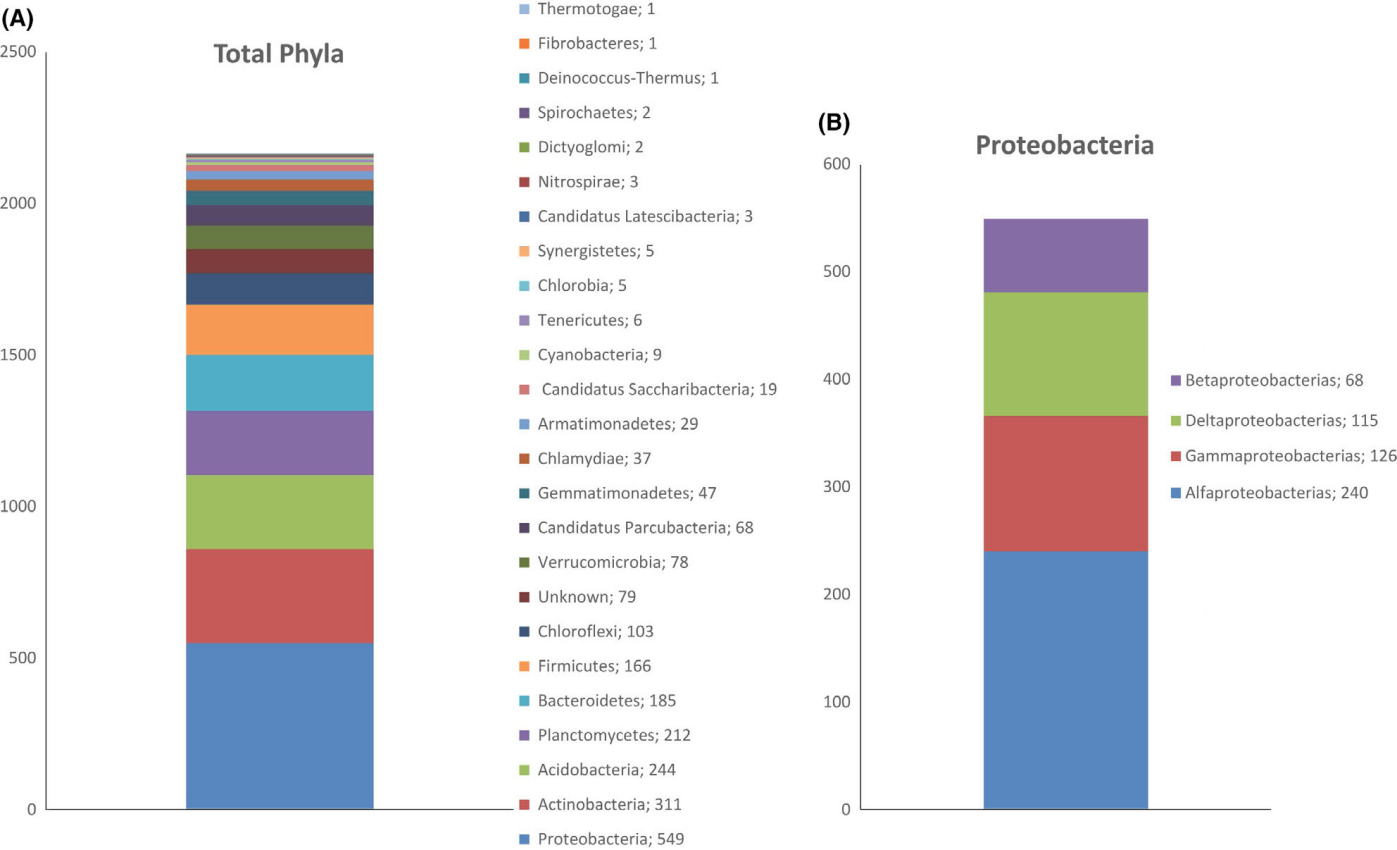
A. Number and proportional distribution of the 2165 bacterial OTUs representing all the detected taxonomic phyla. B. Classes of the phylum Proteobacteria and their number and proportional distribution. Assignment to phyla and classes was based on the Gold database of the Ribosomal Database Project.

The 2165 bacterial OTUs were classified into 24 phyla and 381 genera. Most of the OTUs were identified at the genus level; however, identification down to the species level was impossible owing to database limitations. The soil microbial community was dominated by Proteobacteria, which represented 25% of the bacterial community, followed by Actinobacteria (14%), Acidobacteria (11%), Planctomycetes (10%), Bacteroidetes (9%), Firmicutes (8%), Chloroflexi (5%) and Verrucomicrobia (4%). Less representative phyla (up to 9% of the OTUs) had a lower dominance; 4% of OTUs were not identified. The total number and proportional distribution of OTUs among different phyla are shown in Fig. 1. Within the phylum Proteobacteria, up to 44% belonged to the class Alphaproteobacteria, 23% were Gammaproteobacteria, 21% were Deltaproteobacteria and 12% belonged to the class Betaproteobacteria (Fig. 1). Genera with the highest OTU richness are presented in Table 1.

**Table 1:** Bacterial genera detected in soil cores with the highest OTU richness (number of OTUs) within the most representative phyla

Phylum	Genus	Number of OTUs
Proteobacteria	*Azospirillum*	27
Proteobacteria	*Legionella*	22
Proteobacteria	*Sphingomonas*	21
Actinobacteria	*Conexibacter*	58
Actinobacteria	*Aciditerrimonas*	38
Actinobacteria	*Solirubrobacter*	14
Actinobacteria	*Actinoplanes*	14
Acidobacteria	GP3	67
Acidobacteria	GP1	62
Planctomycetes	*Gemmata*	71
Planctomycetes	*Zavarzinella*	41
Planctomycetes	*Singulisphaera*	34
Bacteroidetes	*Terrimonas*	22
Bacteroidetes	*Solitalea*	18
Firmicutes	*Paenibacillus*	13
Firmicutes	*Bacillus*	13
Chloroflexi	*Ktedonobacter*	57
Chloroflexi	*Thermogemmatispora*	20
Verrucomicrobia	*Opitutus*	12

### Relationship between the abundance of bacterial communities and *B. edulis* sporocarp production and extraradical *B. edulis* mycelium in the soil

To determine relationships between bacterial abundance and *B. edulis* sporocarp production and extraradical mycelium in the soil, linear regressions were carried out for every phylum. For sporocarp production, we observed a statistically significant, but weak positive correlation for the relative abundance of the phylum Verrucomicrobia (*R* ^2^ = 0.20, *P *=* *0.01), and weak negative correlations for the phyla Actinobacteria (*R* ^2^ = 0.20, *P *=* *0.01) and Planctomycetes (*R* ^2^ = 0.14, *P *=* *0.03).

However, the relative abundance levels of the bacterial phyla did not correspond significantly to the amount of extraradical *B. edulis* mycelium in the soil. Only the abundance of Planctomycetes showed a nearly significant negative correlation with the amount of extraradical *B. edulis* mycelium in the soil (*R* ^2^ = 0.13, *P *=* *0.068).

### Impact of site history and fire prevention treatments on bacterial communities

**Figure 2:**
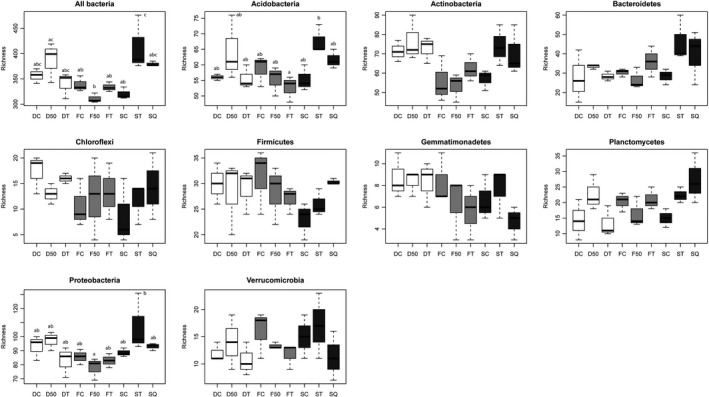
Effect of forest fire prevention treatments on the richness (number of OTUs) of bacterial phyla. Site history (*n *=* *3): F, young burnt; D, young clearing; S, senescent. Treatments: C, control; 50 and T, partial and total clearing; Q, controlled burning. Different letters above the bars within each phylum indicate a statistically significant difference in richness among treatments.

The bacterial richness was significantly affected by the site history of the stand (*P *=* *0.039). Mean OTU richness was highest in the senescent plots (371) and lowest in the young burnt plots (328), and the difference between them was significant (*P *=* *0.002). There was also a significant difference between the richness in the young burnt plots and the young cleared plots (361) (*P *=* *0.013). The different fire prevention treatments only had a significant effect on bacterial richness in the senescent plots, where bacterial richness was significantly higher in plots with total clearing than in the control (*P *=* *0.008). Analysing the effects of the different treatments on the richness of each phylum revealed substantial differences regarding their responses to treatments at sites with different site histories. However, the richness of almost all phyla tended to increase in senescent plots after either total clearing or controlled burning treatments. The richness of Acidobacteria (*P *=* *0.032) and Proteobacteria (*P *=* *0.040) was significantly affected by the treatments (Fig. 2). Both these phyla showed a peak in OTU richness in senescent plots that had been totally cleared.

**Figure 3:**
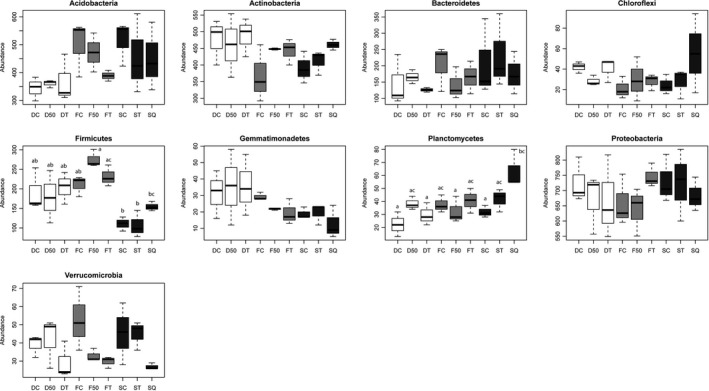
Effect of forest fire prevention treatments on the abundance (number of sequences) of bacterial phyla. Site history (*n *=* *3): F, young burnt; D, young clearing; S, senescent. Treatments: C, control; 50 and T, partial and total clearing; Q, controlled burning. Different letters above the bars within each phyla indicate a statistically significant difference in richness among treatments.

The treatments significantly affected the relative abundance levels of Planctomycetes (*P *=* *0.003). The only significant difference was in the senescent plot where a controlled burning had been performed, and these plots showed the highest abundance (Fig. 3).

**Figure 4:**
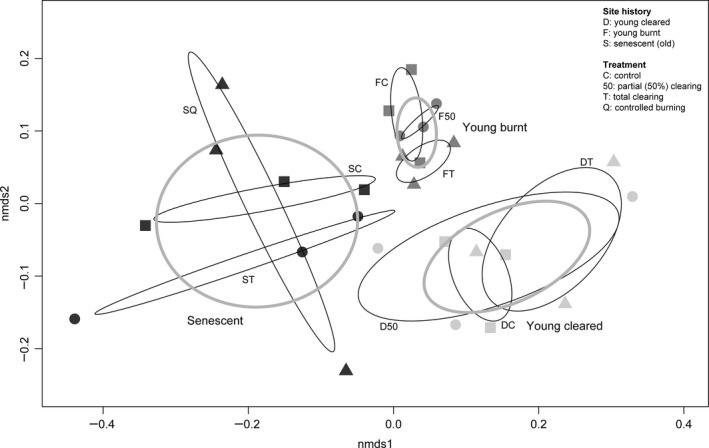
Non‐metric multidimensional scaling (NMDS) ordination plot of bacterial community composition among sites differing in site history and treatment. Site history: F, young burnt; D, young clearing; S, senescent. Treatments: C, control; 50 and T, partial and total clearing; Q, controlled burning.

The NMDS analysis resulted in a two‐dimensional solution with a final stress value of 0.0693. The ordination plot revealed a strong structuring of bacterial communities according to the site history and, to a lesser extent, to the treatment (Fig. 4). MRPP confirmed the importance of the site history in shaping bacterial community composition (effect size A = 0.0907, probability *P *<* *0.00001). Similarly, PERMANOVA indicated that bacterial community composition differed significantly among the different site histories (*P *=* *0.0002).

Our analysis to identify bacterial indicator species associated with the most productive *B. edulis* sites revealed 18 OTUs that were characteristic of plots with high *B. edulis* sporocarp production (Table 2), including members of the *Azospirillum*,* Gemmatimonas* and *Opitutus* genera.

**Table 2:** Bacterial OTUs considered to be significant indicators of highly productive plots of *Boletus edulis* sporocarps

OTU	*P*	Accession	%^a^	bp^b^	Name^c^	Phylum^c^
OTU 627	0.004	DQ768106	94.7	245	*Archangium*	Proteobacteria
OTU 1240	0.0094	AJ229235	95.9	245	*Opitutus*	Verrucomicrobia
OTU 1741	0.0098	FN400860	97.6	245	*Mucilaginibacter*	Bacteroidetes
OTU 1541	0.0114	AJ229235	93.1	245	*Opitutus*	Verrucomicrobia
OTU 2893	0.0114	EF457384	97.1	245	Gp1	Firmicutes
OTU 471	0.0162	AJ535711	90.2	245	*Stella*	Proteobacteria
OTU 7	0.0214	FN400860	99.2	245	*Mucilaginibacter*	Bacteroidetes
OTU 501	0.0256	AB072735	91.0	245	*Gemmatimonas*	Gemmatimonadetes
OTU 402	0.0292	CP001854	95.9	245	*Conexibacter*	Actinobacteria
OTU 449	0.0294	AY324110	91.8	245	*Azospirillum*	Proteobacteria
OTU 2331	0.0324	AB072735	92.7	245	*Gemmatimonas*	Gemmatimonadetes
OTU 3	0.0342	HM214537	96.3	245	*Terriglobus*	Acidobacteria
OTU 2324	0.0358	X97098	95.5	245	Gp1	Firmicutes
OTU 279	0.0372	AJ401115	92.2	245	Subdivision3_genera_incertae_sedis	Verrucomicrobia
OTU 602	0.0384	FJ177532	94.7	245	*Ferruginibacter*	Bacteroidetes
OTU 2943	0.0392	EF457390	98.0	245	Gp1	Firmicutes
OTU 3279	0.0412	EF457407	100	245	Gp4	Firmicutes
OTU 1543	0.0454	AJ229235	90.2	245	*Opitutus*	Verrucomicrobia

**a.** %: sequence similarity.

**b.** bp: pairwise alignment length.

**c.** Name and phylum of the most similar sequence in the Gold database, part of the Ribosomal Database Project.

## Discussion

The phyla Proteobacteria, Actinobacteria and Acidobacteria were the most abundant, which support the findings reported in previous studies of forest soils (Deveau *et al*., 2016; Sun *et al*., 2017). These phyla seem to be abundant in most soils (Lauber *et al*., 2009), which appears to indicate their functional importance (Lladó *et al*., 2017). Alphaproteobacteria represented the most prevalent class within Proteobacteria. This finding could be explained by the low pH of the soils at the study site. pH is an important driver of bacterial community composition (Lauber *et al*., 2009), and acidic soils are usually dominated by Alphaproteobacteria and Acidobacteria (Felske *et al*., 1999; Kaiser *et al*., 2001; Baldrian *et al*., 2012; Shen *et al*., 2013). Some members of the class Alphaproteobacteria mediate N_2_ fixation (Lladó *et al*., 2017), strongly influencing the abundance of ectomycorrhizal fungi (Lauber *et al*., 2008). The Alphaproteobacterial genera *Sinorhizobium* and *Rhizobium*, present in the studied soil, both harbour multiple nitrogen fixer species (Barbieri *et al*., 2005).

Regarding the correlation analysis between bacterial abundance and *B. edulis* sporocarp production, some previous studies have shown that although interactions between bacteria and fungi can be crucial (Boer *et al*., 2005), these interactions can be positive, negative or neutral, i.e. they can involve pathogenic or helper bacteria as well as mutualists (Bonfante and Anca, 2009; de Boer, 2017). In our study, the phylum Verrucomicrobia showed a weak positive correlation with the production of sporocarps. Buckley and Schmidt (2001) suggested that Verrucomicrobia abundance may increase with soil moisture content. At our study sites, an increase in shrub cover increased soil moisture in the soil and was associated with increased *B. edulis* sporocarp production. There was a suggested negative correlation for Actinobacteria which could be explained by its heliophilous behaviour. The abundance of these bacteria tends to increase in the absence of vegetation, a trend that we observed in the treatments where the host plants were removed. This response was observed by Zhang *et al*. (2016) who reported a significant increase in these bacteria after forest clear‐cutting. By contrast, the abundance of *B. edulis* mycelium in the soil, and consequently sporocarp production, tends to decrease when the host is eliminated (Mediavilla *et al*., 2017).

When analysing the impact of fire prevention treatments on bacterial communities, most of the phyla did not show a significant response in terms of richness and abundance. This suggests a certain resilience of the bacterial communities after forest management treatments and supports the results of Smith *et al*. (2008) who highlighted the important role of bacteria in the resilience of forests to disturbances and in the regeneration processes. Other forest management practices have also been reported to have a minor impact on soil bacterial community structure and diversity (Nacke *et al*., 2011). The dominant vegetation and land use were reported to affect fungi more strongly than bacteria, especially the ectomycorrhizal taxa because of their dependence on the host plant (Buée *et al*., 2009; Zinger *et al*., 2011).

Both total clearing and controlled burning increased bacterial richness, which may be explained by the increase in stand heterogeneity. Senescent *C. ladanifer* stands normally have a high density of the plants reaching almost total cover (Hernández‐Rodríguez *et al*., 2013), which provides a rather homogeneous structure with a low diversity of niches. Both the total clearing and the controlled burning treatments essentially removes most of the closed scrublands, creating new available niches microorganisms. At this point, any perturbation would be desirable in order to increase the bacterial richness (Santillán *et al*., 2018).

In terms of proportional abundance, the only bacterial phylum that was affected by the fire prevention treatments was the Planctomycetes, which appeared to be more abundant in the senescent plots where the controlled burning was performed. It was previously observed that some years after prescribed burning, nutrient levels and mineralization rates decrease in the affected soils (Choromanska and DeLuca, 2001; Reich *et al*., 2001; Carter and Foster, 2004). In these conditions, Planctomycetes could survive since their members are typically slow‐acting decomposers (Dedysh and Kulichevskaya, 2014).

The young burnt plots displayed the lowest bacterial richness. This could be because most of the phyla detected at the study sites prefer acid soils. However, the pH in the young burnt plots was likely higher than in the other plots because fire tends to increase soil pH (Shen *et al*., 2016) due to the deposition of ash (Certini, 2005). Our findings agree with those of Smith *et al*. (2008), who reported that bacterial communities tend to recover faster in cleared sites than in burned ones.

When analysing bacterial composition, we emphasize the strong influence of the stand site history in our study. Bonfante and Anca (2009) also reported that bacterial community composition is determined by site history. This also supports the findings of Smith *et al*. (2008), who reported the effect of harvesting and fires on the composition of forest soil microbial communities. Fires drive a shift in the bacterial community structure (Rousk *et al*., 2010) due to the increase of pH. Baath *et al*. (1995) also reported that an increase in soil pH due to fire had a noteworthy effect on bacterial community composition. Specifically, we observed a higher abundance of Firmicutes in the young plots, which were most abundant in plots established after a wildfire. This finding supports those reported by Smith *et al*. (2008), who also reported that Firmicutes were characteristic in post‐fire soils. This phylum is able to survive under extreme conditions and may be favoured by wildfires.

Our results revealed some bacterial OTUs that could be considered as indicator species of the most productive *B. edulis* sites. Of these, the genus *Azospirillum* represents the best‐characterized genus containing plant growth‐promoting rhizobacteria (Steenhoudt and Vanderleyden, 2000). Its members possess a number of beneficial properties, including nitrogen fixation, production of indole‐3‐acetic acid (IAA) and deamination of the ethylene precursor 1‐aminocyclopropane‐1‐carboxylate (ACC) (Creus *et al*., 2005; Blaha *et al*., 2006). Furthermore, plants associated with *Azospirillum* communities develop an increased number of lateral roots and root hairs, which will enhance not only the amount of root surface area available for nutrient uptake (Steenhoudt and Vanderleyden, 2000) but also the number of mycorrhizal roots. We also identified the genus *Gemmatimonas* as an indicator species, which is considered to be a phosphate‐solubilizing bacterium (Yang *et al*., 2017) and has also been listed among N_2_‐fixing bacteria (Krieg *et al*., 2010). The genus *Opitutus* was the most predominant among the indicator species. This genus belongs to the phylum Verrucomicrobia, from which we obtained a positive correlation with *B. edulis* sporocarp production, which could be due to their role in C cycling (Fierer *et al*., 2013).

Based on the results obtained in this study, it is possible to suggest some management directions. Because senescent stands tend to have lower bacterial richness, perturbation resulting in the rejuvenation of these areas are desirable, to trigger an increase in bacterial richness. This fact will also allow for an increase of *B. edulis* sporocarps production and for a decrease in potential fire severity by reducing the amount of available fuel. In this context, some mechanical management (i.e. clearing) seems to be the best option, because its effect on bacterial communities is less severe than in the case of burning, and this treatment showed favourable effects on bacterial richness in our study. However, it is desirable to maintain habitat heterogeneity at the landscape scale by leaving patches of senescent scrublands intact to maximize overall bacterial richness in the area.

Here we present insights into the bacterial communities associated with highly productive *B. edulis* sites in *C. ladanifer* scrublands under different fire prevention treatments, using a metagenomics approach. We demonstrated that the site history influenced the bacterial composition and identified bacterial species that were indicators of highly productive *B. edulis* sites, noting the biological role played by some bacterial phyla. Despite these findings, there is still much uncertainty about the association between individual bacterial species and fungal fruiting. Although further research is needed in this field, this study provides a better understanding of the triple symbiosis (plant–fungus–bacteria) involved in *C. ladanifer*–*B. edulis* systems.

## Materials and methods

### Study site

The study area was located in Zamora province, north‐western Spain (0730462–0731929 longitude‐UTM, 4619644–4621757 latitude‐UTM 29T Grid), 750–780 m above sea level. Twenty‐seven plots (2 × 50 m) were established at the study site. The characteristic natural community is dominated exclusively by *C. ladanifer,* with ca. 80% canopy coverage. This study site has been widely used as an experimental area to study the effects of fuel reduction treatments on fungal communities (Hernández‐Rodríguez *et al*., 2013, 2015b, 2017).

Based on the soil characteristics, the soil in this zone is classified as Inceptisol suborder Xerept (Soil Survey Staff, 2010) and characterized by stoniness, acidity (pH 5.0–5.5), and lack of calcium (1.5–4.0 meq 100 g^−1^, atomic absorption method) and phosphorus (< 4.0 mg kg^−1^, Olsen method). The availability of nitrogen (0.09–0.18 g 100 g^−1^, modified Kjeldahl method) and potassium (71–128 mg kg^−1^, atomic emission method) is variable, with a good level of humification. This region is characterized by a sub‐Mediterranean climate: mean temperatures range from 14.5 to 15.8°C, and annual precipitation ranges from 450 to 700 mm. Climatic data were provided by the closest meteorological station (Alcañices, 0724617 Longitude‐UTM, 4618218 Latitude‐UTM, 29T Grid and 806 m above sea level, Spanish Meteorological Agency).

### Fuel reduction treatments

The study site comprised three areas with different ages and site histories: (a) an eight‐year‐old stand regenerating from a wildfire (young burnt); (b) an eight‐year‐old stand following total clearing of the previous stand (young cleared); and (c) a 20‐year‐old senescent stand following a natural wildfire (senescent). Because of the short cycle life of *C. ladanifer*, a stand of 20 years of age is considered as senescent or declining (Oria‐de‐Rueda *et al*., 2008). These three stands are located in the same area of *C. ladanifer* scrubland and share similar mesoclimatic and edaphic conditions. Specifically, young burnt and young cleared areas are around two hundred metres apart, while the senescent area is a kilometre away. More information on the experimental setup can be found in Mediavilla *et al*. (2017).

Forest fire prevention treatments were applied depending on their feasibility in accordance with the age of the stands and vegetation characteristics. In the eight‐year‐old stands (i) and (ii), the fuel reduction treatments were as follows: (1) uncleared, which acted as the control; (2) 50% cleared; and (3) total clearing, i.e. all *C. ladanifer* plants were removed. In the 20‐year‐old stand (iii), the treatments were as follows: (1) uncleared, which acted as the control; (2) total clearing; and (3) controlled burning (Table 3).

**Table 3:** Forest fire prevention treatments applied in accordance with the plant age and site history of the stands

Plant age	Site history	Areas	Treatments
8 years old	Wildfire	Young burnt	Control (uncleared)
	Total clearing	Young cleared	50% cleared
			Total clearing
20 years old	Wildfire	Senescent	Control (uncleared)
			Controlled burning
			Total clearing

Total clearing was performed using a tractor with a brush thrasher mower; whereas, the 50% cleared treatment was carried out manually by removing half of the plants with a brush cutter. Both clearing treatments were carried out in spring 2010. Controlled burning was performed with the help of Zamora EPRIF (Integral Fire Prevention Team) (Ministry of Agriculture, Food and Environment) in October 2010 under favourable weather conditions that allowed ignition without the risk of uncontrolled fire. For each treatment, we collected samples from three different plots. In summary, we studied three different stands with three different forest fire prevention treatments per stand, with three plots per treatment, resulting in a total of 27 plots sampled.

### Sampling and molecular work

At each site, in December 2013, five soil cores, 3.5 cm in diameter and 26 cm in length, were extracted at 5 m intervals along the longitudinal axis of the plot (Taylor, 2002). The soil was dried at room temperature, and coarse elements were discarded. The five cores were pooled resulting in a composite soil sample per site. DNA was extracted from 0.25 g of dry soil per sample using a PowerSoil^®^ DNA Isolation Kit (MoBio Laboratories, Carlsbad, CA, USA) and according to manufacturer's instructions. The primers 515F and 806R‐trP1 (Caporaso *et al*., 2011, 2012) were used to amplify a portion of ca. 255 bp of bacterial 16S rDNA. The forward primer was labelled with sample‐specific multiplex identification DNA‐tags (MIDs). For each of the 27 samples, the following PCR protocol was used for three positive and one negative reaction: one cycle of 95°C for 5 min, then 35 cycles of 95°C for 20 s, 54°C for 30 s, and 72°C for 1.5 min, ending with one cycle of 72°C for 7 min. Negative PCR reactions were performed for each primer pair and contained elution buffer instead of DNA. PCR products were checked for DNA concentrations using QIAxcel Advanced System (Qiagen, Venlo, The Netherlands). Emulsion PCR and Ion Torrent sequencing was carried out at the Naturalis Biodiversity Center (Leiden, The Netherlands). We used the sequencing Ion 318™Chip to allow for the highest possible sequencing coverage.

### Quantification of *B. edulis* mycelium in the soil and sporocarp production levels

*Boletus edulis* DNA was amplified using real‐time PCR (qPCR) in an Applied Biosystems^®^ 7500 Real‐Time PCR System (Applied Biosystems, Mannheim, Germany), using the kit qPCR *Boletus edulis*‐VK provided by Vacunek S.L. (Bilbao, Spain), and according to the manufacturer's instructions and a final reaction volume of 25 μl. The kit provides the necessary reagents and enzymes, mixed in a single master mix. The kit uses a Taqman probe marked with FAM‐BHQ1^®^ and also a positive internal control with primers and a Taqman probe marked with JOE‐BHQ1^®^, which allows for the detection of false negatives caused by inhibition. Real‐time PCR cycling conditions were 10 min at 95°C, 45 cycles at 95°C for 15 s and 60° for 60 s. Three replicates of each sample were included in the analysis, as well as a negative control (using deionized water instead of DNA template). A standard curve with five points and three replicates per point using known amounts of mycelium in the soil was generated. This curve was used to translate the outputs from the qPCR System into amount of mycelium in soil. Ten‐fold serial dilutions were performed from 11 400 000 to 1140 ng of mycelium g of soil^−1^. See Mediavilla *et al*. (2017) for more detailed information. To quantify the production of *B. edulis* sporocarps, all *B. edulis* sporocarps were collected and weighed on a weekly basis during the mushroom season from 2010 to 2013. The sporocarps were harvested, transported to the laboratory and stored at 4°C. Fresh weight was measured (kg fw ha^−1^ year^−1^) within 24 h after collection (Hernández‐Rodríguez *et al*., 2015b).

### Bioinformatic analysis

The initial clean‐up of the raw sequence data was carried out using the online platform Galaxy (https://main.g2.bx.psu.edu/root), in which the sequences were sorted into samples, and identification tags were removed. Poor‐quality ends were trimmed off based on a 0.02 error probability limit in geneious pro 5.6.1 (BioMatters, Auckland, New Zealand). Subsequently, sequences were filtered using usearch v.8.0 (Edgar, 2010) based on the following settings: all sequences were truncated to 200 bp and sequences with an expected error of > 1 were discarded. For each sample, sequences were collapsed into unique sequence types while preserving their counts and excluding singletons. The quality‐filtered sequences from all samples were grouped into operational taxonomic units (OTUs) at 97% sequence similarity and putative chimeric sequences were removed using USEARCH. We assigned sequences to taxonomic groups of bacteria based on pairwise similarity searches against the curated Gold database of the Ribosomal Database Project (Cole *et al*., 2009). The resulting matrix containing only OTUs with at least 70% sequence similarity to a bacterial reference sequence had 105 553 quality‐filtered sequences. We rarefied the OUT matrix to the smallest sequence count per sample (2342 sequences), and the resulting matrix of 2165 OTUs was used for all subsequent statistical analyses.

Representative sequences of bacterial OTUs were submitted to GenBank with the accession numbers MK323080 – MK325185.

### Statistical analysis

We compared the richness and abundance of bacterial phyla among the sampling sites using ANOVA and Tukey's HSD test in r (R Development Core Team 2016). Furthermore, linear regression analyses in r were used to examine relationships between the abundance of bacterial phyla and sporocarp production, as well as the extraradical *B. edulis* mycelium in the soil variable (logarithm of *B. edulis* mycelium concentration) as quantified by real‐time PCR (Mediavilla *et al*., 2017). To compare bacterial community composition across the studied stands, we ran non‐metric multidimensional scaling (NMDS) analysis on the Hellinger‐transformed OTU table. Two ordinations were carried out, one based on abundance and another based on presence/absence. Data were subjected to 999 iterations per run using the Sørensen similarity (Bray–Curtis index) and a random number to start. In addition, we tested whether bacterial communities were statistically different across forest types using the multiple‐response permutation procedure (MRPP) and permutation‐based nonparametric MANOVA (PERMANOVA) (Anderson, 2001). Finally, we tested whether individual OTUs showed a significant association with the sites with the highest *B. edulis* sporocarp production levels using indicator species analyses (Dufrêne and Legendre, 1997) in PC‐ORD. For this purpose, the plots were classified into two categories according to their *B. edulis* sporocarp production values: plots producing 0–20 kg ha^−1^ were considered to be poorly productive and those producing > 20 kg ha^−1^ were considered to be highly productive (Hernández‐Rodríguez *et al*., 2015a).

## Conflict of interest

None declared.
